# Effects of dropping out of dental treatment on the oral health-related quality of life among middle-aged subjects using web research

**DOI:** 10.1371/journal.pone.0205462

**Published:** 2018-10-31

**Authors:** Tomotaka Kato, Yojiro Umezaki, Toru Naito

**Affiliations:** Section of Geriatric Dentistry, Department of General Dentistry, Fukuoka Dental College, Fukuoka, Fukuoka-prefecture, Japan; University Lyon 1 Faculty of Dental Medicine, FRANCE

## Abstract

**Objective:**

The oral health-related quality of life has recently been reported to be a rather important aspect of general health. Dropping out of dental treatment has long been a problem plaguing oral health. However, the relationship between dropout for dental treatment and the oral health-related quality of life is unclear. The purpose of this study was to investigate the oral health-related quality of life in patients who dropped out of dental treatment.

**Materials and methods:**

We conducted a questionnaire-based investigation using web research. The participants were allocated to two groups (dropout group and maintenance group). The dropout group included participants who had stopped visiting their dental office in the past and had not revisited in the last decade. The maintenance group included patients who visited their dental office continually for a regular checkup. We analyzed the General Oral Health Assessment Index (GOHAI) as an indicator of the oral health-related quality of life and assessed the background characteristics of the subjects.

**Results:**

We analyzed 225 people in the dropout group and 236 people in the maintenance group. The score of GOHAI was significantly different between the 2 groups (dropout group:47.07, maintenance:48.97, *p* = 0.035), and the more frequent dropouts brought the less GOHAI score (*p* = 0.012). Furthermore, the results of a logistic regression analysis showed that dropping out of dental treatment was significantly associated with the GOHAI score (*p* = 0.002).

**Conclusion:**

A relationship was demonstrated between the oral health-related quality of life and dental treatment dropout. Furthermore, dental treatment dropout seemed to have negative effects on the oral health-related quality of life.

## Introduction

According to current understanding, periodontal disease has a relationship with systemic diseases [1–4]. It is therefore very important to control periodontal diseases for not only oral health but also general health. For controlling periodontal disease, continuing treatment is very important as supportive periodontal therapy (SPT) [5,6]. On the other hand, dental treatment dropout is one of the strongest risk factors influencing periodontal disease progression [7]. However, only 20%-50% of people [8], and many patients drop out from visiting dental clinic [9]. A decline in the oral health of these dropout patients is therefore of substantial concern and dropping out of dental treatment is a weighty issue. However, it is difficult to perform an analysis of patients who have dropped out of dental treatment, as they do not visit dental clinic or undergo examinations.

Oral health is reported to be a very important part of one’s well-being according to the World Health Organization [10], and the oral health-related quality of life shares a relationship with the general quality of life [11,12]. Several studies have explored ways to measure the oral health-related quality of life. The General Oral Health Assessment Index (GOHAI) [13] has been developed as one such method and is easy to use because of the self-reported aspect of its measurement [14]. In addition, the GOHAI is reported to be significantly associated with the oral health status [15–17], socioeconomic status [18], nutritional status [19], and psychological status [20,21]. However, there have been no reports on the relationship between the GOHAI and dropping out of dental treatment, hence the effects of dental treatment dropout on the oral health-related quality of life are unclear. Therefore, we made a hypothesis that dropping out of dental treatment decreased oral health-related quality of life. The purpose of this study was to clarify the relationship between the oral health-related quality of life and dropping out of dental treatment using web research.

## Methods

### Participants and ethical considerations

This study was conducted using web research. Participants who enrolled with an internet research company (JUSTSYSTEMS CORPORATION, Tokyo, Japan) on January 20–24, 2017. According to the Survey of Dental Diseases by Japanese Ministry of Health, Labour and Welfare [22], more than half of over 45 years people have periodontal disease (existing periodontal pocket >4mm). We therefore set up that over 45 years of age were administered a questionnaire evaluation. The subjects were allocated to two groups: the “dropout” group and the “maintenance” group. The “dropout” included participants who had stopped visiting their dental office in the past and had not revisited in the last 10 years. The “maintenance” included patients who visited their dental office continually for a regular checkup one or more times in the past year. Both participants were evaluated each groups with self-reported on website. Participant consent was also provided on the web, and we set the questionnaires that the only agreed participants could answer on website.

This study was approved by the Ethics Committee for Clinical Research at Fukuoka Gakuen (approval number 324). The research company provided analysts with the results of the questionnaire only, and the analysts had no access to subjects’ individual information.

### Sample size

The sample size was calculated by comparing the average between the two groups, with the α error set to 0.05 and the power to 0.95. A previous study showed that the Δ was 1.5 and the standard deviation 3.5 [23]. We therefore planned to include over 220 subjects in each group.

### Background characteristics of the participants

The following background characteristics of the participants in both groups were analyzed: age, gender, marriage status, and child status. These items were based on those described in a previous study. The number of frequency for dropout from dental visiting was counted in the “dropout”.

### The assessment of the oral health-related quality of life

The Japanese version of the GOHAI was used to analyze the oral health-related quality of life [14]. Participants were asked to respond using a 5-point scoring scale (always, often, sometimes, seldom and never) regarding the presence of the 12 question items of the GOHAI over the preceding 3 months. The GOHAI comprises three domains of oral health; “physical functions” including eating, speech and swallowing; “psychosocial functions” including worries or concerns about oral health; and “pain or discomfort” including teeth or gums sensitive, pain relief medication, and feeling discomfort when eat anything. The total GOHAI scores range from 12 to 60. A high GOHAI scores indicated a positive self-perception of oral health, while lower scores indicated poorer oral health and reflected oral health problems. In the present study, we analyzed participants with GOHAI-high score for logistic regression models. The median value of the GOHAI national norm for Japanese individuals was 55.0. Therefore, we defined the participants with GOHAI scores of ≥ 55 as the GOHAI-high group.

### Statistical analyses

Statistical analyses were performed using the IBM SPSS Statistics software program (Ver. 22.0; International Business Machine Japan, Tokyo, Japan). In all analyses, the significance was set at *p* < 0.05. Mann-Whitney U test was used to compare the background characteristics and the GOHAI score between the “maintenance” and “dropout”. The total GOHAI score was analyzed with the Kruskal Wallis test on the differences about numbers of dropout dental visiting.

## Results

### Characteristics of participants

The characteristics of the study subjects are shown in Table 1. We finished recruiting subjects with 225 people in the “dropout” and 236 people in the “maintenance”. No statistically significant differences were noted between the “dropout” and “maintenance” in the sex, marriage status, and child status.

**Table 1:** A comparison of the characteristics between the dropout and maintenance groups.

	Dropout	Maintenance	*P* value
Number of subjects	225	236	
Male/Female	115/110	123/113	0.83
Age (years)	56.3 ± 7.0	56.6 ± 7.4	0.37
Married/Single	177/48	188/48	0.79
With/without children	165/60	160/76	0.19

Mann-Whitney *U* test

### Differences in the GOHAI between groups

The comparison of the GOHAI total scores for each of the 12 items and 3 domains between the “dropout” and “maintenance” were shown in Table 2. Significant differences were noted between the two groups in the GOHAI total score using the Mann-Whitney U test (*p* = 0.005). Each of the three domains also showed significant differences, and 10 items were also noted significant differences between the two groups.

**Table 2:** GOHAI data obtained from dropout group and maintenance group.

	Dropout	Maintenance	*P* value
Physical domain	15.64 (3.24)	16.19 (3.56)	0.018
1.How often did you limit the kinds or amounts of food you eat because of problems with your teeth or denture?	3.82 (0.94)	3.98 (1.01)	0.041
2.How often did you have trouble biting or chewing any kinds of food, such as a firm meat or apples?	3.67 (1.02)	3.75 (1.15)	0.19
3.How often were you able to swallow comfortably?	4.07 (0.85)	4.21 (0.96)	0.031
4.How often have your teeth or dentures prevented you from speaking the way you wanted?	4.08 (0.90)	4.25 (0.99)	0.009
Psychosocial domain	19.79 (4.19)	20.70 (4.43)	0.005
6.How often did you limit contacts with people because of the condition of your teeth or dentures?	4.21 (0.86)	4.36 (0.89)	0.026
7.How often were you pleased or happy with the appearance of your teeth, gums or dentures?	3.67 (1.11)	3.96 (1.12)	0.003
9.How often were you worried or concerned about the problems with your teeth, gums or dentures?	3.7 (1.06)	3.87 (1.14)	0.041
10.How often did you feel nervous or self-conscious because of problems with your teeth, gums or dentures?	4.08 (0.93)	4.24 (0.98)	0.029
11.How often did you feel uncomfortable eating in front of people because of problems with your teeth or dentures?	4.12 (0.92)	4.27 (0.95)	0.043
Pain/discomfort domain	11.64 (2.27)	12.08 (2.74)	<0.001
5.How often were you able to eat anything without feeling discomfort?	3.96 (0.89)	4.08 (1.11)	0.018
8.How often did you use medication to relieve pain or discomfort around your mouth?	4.16 (0.93)	4.25 (1.01)	0.152
12.How often were your teeth or gums sensitive to hot, cold or sweet foods?	3.52 (1.05)	3.75 (1.11)	0.022
Total score	47.07 (9.06)	48.97 (10.20)	0.005

Mann-Whitney *U* test

### Relationship between the total score and number of frequency for dropout experiences

Participants were divided into four groups according to the number of times that they have dropped out from dental treatment. (0, 1–2, 3–5, and >5). Significant differences were noted among the four groups using the Kruskal Wallis *H* test (Table 3). A low GOHAI score was shown to be associated with a high number of dropouts.

**Table 3:** The GOHAI average score with difference in the number of treatment dropouts.

	Maintenance (0 dropout)	1–2 dropouts	3–5 dropouts	More than 5 dropouts
GOHAI (SD)	48.98 (10.20)	47.50 (8.89)	47.29 (8.99)	43.70 (9.97)

Kruskal Wallis *H*-test *p =* 0.0123

### Logistic regression analyses of predictors for inclusion in the GOHAI-high group

Dropping out of dental treatment (≥1 dropout experiences) and sex were shown to be factors significantly associated with inclusion in the GOHAI-high group (Table 4). The odds ratio for dropping out of dental treatment was 0.516 (95% confidence interval 0.340–0.785). The Hosmer-Lemeshow test value of this model was *P* = 0.103, while the identification rate was 69.8%.

**Table 4:** Logistic regression of the predictors of the GOHAI high-group.

	*β*	Odds ratio	(95% CI)	*P* value
Sex	0.663	1.941	(1.083–3.480)	0.026
Age	-0.018	0.983	(0.951–1.015)	0.285
Married	0.095	1.100	(0.581–2.082)	0.769
Children	-0.126	0.881	(0.510–1.524)	0.651
Occupation				0.555
Residence				0.086
Treatment dropout	-0.661	0.516	(0.340–0.785)	0.002

## Discussion

In the present study, people in the “dropout” were seemed significantly lower oral health-related quality of life than people in the “maintenance”, and the more frequent dropouts brought the less GOHAI score. Moreover, dropping out of dental treatment was significantly associated with the GOHAI score by logistic regression analysis. Therefore, it was consider that to prevent dropout dental treatment might be important to prevent decreasing of oral health-related quality of life.

Several previous reports have described the effects of periodontal therapy on the oral health-related quality of life [24,25]. However, none have focused on the effects of dental treatment dropout on the oral health-related quality of life. To our knowledge, this is the first study to demonstrate the relationship between the oral health-related quality of life and dropping out of dental treatment. The fact that the relevant subjects would not visit dental clinics or undergo dental examinations no doubt made it difficult to perform analyses.

Our study showed that dental treatment dropout was associated with a low oral health-related quality of life. No similar studies have been performed, but some reports have shown that undergoing dental treatment is associated with a high oral health-related quality of life [26]. Therefore, our results were reasonable based on these previous reports. However, our study showed that the oral health-related quality of life significantly varied by gender. The oral health-related quality of life is known to be affected by many factors [14], but no marked differences by gender have been noted in previous reports on the development of assessment indices using this study [14,27]. However, some studies have reported that gender affected[28,29] the oral health-related quality of life. For example, female have shown a tendency to have a low oral health-related quality of life [28]. These results showed the same tendency as in the present study. It was not easy to clear the reason of gender differences, but there were some helpful reports to explain the gender differences by self-administered questionnaire[30,31]. The study about sleep quality reported that female were lower sleep quality than men[30], and the study of pain threshold level reported that female were lower pain threshold level than men[31]. From the result of these studies, female might feel low well-being themselves, and female might feel low oral health-related quality of life themselves. Further studies should be performed to clarify these gender differences in the oral health-related quality of life.

In this present study, it was seen negative oral health with people in “dropout”, and it was very important to discuss about preventing dropout dental treatment. However, there was no study about preventing dropout dental treatment to our knowledge. On another front, dental diseases were almost lifestyle related diseases, thus it was useful to refer to the preventing dropout with diabetes mellitus. Using mobile applications had effects of decreasing dropouts from diabetic care [32]. Therefore, mobile technologies might have possibilities to prevent dropout dental treatment. However, it was still unclear to prevent dropout dental treatment, and there were limited reports. Therefore, we should conduct further studies to prevent dropout dental treatment.

Our study was limited by its use of a web research platform. Therefore, there were some selection biases. In addition, the questionnaires were also selected by self-certification. However, more than 80% of Japanese people use the Internet recently [33], and inappropriate respondents were removed to improve response reliability in web research products [34,35], and the web research company used by this study also screened monitors routinely. Comparing phone interview study of which response rate was 5.38% in our prior study, web research like this study might be more rational and reliable.

## Conclusion

Our study showed that subjects who dropped out of visiting their dental office had a worse oral health-related quality of life than those who continued to attend visits. Furthermore, dropping out of dental visits seemed to have a negative effect on the oral health-related quality of life.

## Supporting information

S1 Dataset This is the dataset of this study. (XLSX)
